# Using Popular Foods Consumed to Inform Development of Digital Tools for Dietary Assessment and Monitoring

**DOI:** 10.3390/nu14224822

**Published:** 2022-11-14

**Authors:** Juliana Chen, Amanda Grech, Margaret Allman-Farinelli

**Affiliations:** 1 Discipline of Nutrition and Dietetics, Susan Wakil School of Nursing and Midwifery, Faculty of Medicine and Health, The University of Sydney, Camperdown, NSW 2006, Australia; 2 Charles Perkins Centre, The University of Sydney, Camperdown, NSW 2006, Australia

**Keywords:** dietary assessment, diet, nutrition, information technology

## Abstract

Knowing the type and quality of the most popular foods consumed by a population can be useful in the design of technologies for monitoring food intake and interventions. The aim of this research was to determine the most frequently consumed foods and beverages among the Australian population and provide recommendations for progressing the design of dietary assessment technologies. Analysis of the first 24 h recall of the most recent Australian National Nutrition and Physical Activity Survey was conducted. The most popular foods and beverages consumed by energy (kJ) and by frequency were calculated. There were 4515 separate foods and beverages reported by 12,153 people. Overall, the top 10 foods that contributed most energy included full fat milk, beer, white rice, white bread, red wine, cola soft drinks, bananas, red apples, wholewheat breakfast cereal and white sugar. The five most frequently reported foods and beverages were tap water, black tea, full fat milk, instant coffee, and sugar. Understanding the most popular foods and beverages consumed can support innovations in the design of digital tools for dietary surveillance and to reduce under-reporting and food omissions. These findings could also guide the development of more tailored and relevant food databases that underpin these technologies.

## 1. Introduction

Nutrition is a critical modifiable risk factor to prevent chronic diseases, including certain cancers, diabetes, cardiovascular disease and other diseases such as musculoskeletal disorders [1]. Better diet quality is also increasingly linked to mental health, dental health and fertility and can help people look and feel better [2,3,4,5]. Better quality diets can be achieved following a range of dietary patterns such as the Mediterranean diet and the dietary approaches to stop hypertension (DASH) diet, but the individual foods consumed will be similar and from recommended food groups such as fruit, vegetables, wholegrains, legumes, nuts, calcium-rich foods such as low fat dairy and other lean proteins [6]. Other foods and beverages high in sodium, saturated fat, alcohol, and added sugars would be consumed in low quantities or not at all.

National nutrition surveys can provide valuable insights into the dietary patterns and specific foods consumed by a population [7]. However, there is more limited exploration of what specific foods and beverages are frequently consumed by the population. One study using data from the NHANES 2007–2012 has explored the 25 most popular foods consumed by US adults and by specific age groups (19–35 years, 36–55 years, ≥56 years) [8]. The detailed information of foods derived from such large cross-sectional dietary surveys are compiled into databases that include the nutritional composition of the foods [9]. Understanding what foods and beverages are commonly consumed by the population could contribute to greater refinement of these food databases while also avoiding loss of granularity and quality of nutrition information. In turn, these databases can also be incorporated into technologies for monitoring population food intake [10,11,12,13].

Advancements in digital dietary assessment methods and tools, such as smartphone applications (apps), 24 h recall platforms, image-based dietary records, sensors and wearable devices provide the opportunity to increase the efficiency and accuracy of dietary surveillance and decrease the burden associated with data collection [14,15]. Nevertheless, the food databases that underpin apps and self-administered 24 h recall technologies in particular often pose challenges to users including difficulties with finding correct food items when a multiplicity of choices must be scrolled through. Food recording can be too time-consuming, particularly the entry of composite foods and mixed dishes [16,17,18,19]. Increasing the relevance of search returns from the food databases could reduce participant burden, assist selection of the right food and prevent food omissions and under-reporting [12].

Data on popular foods and beverages can be advantageous in guiding search tools or verifying foods and beverages identified by digital dietary assessment methods [8]. Furthermore, inaccuracies in nutrient outputs from these digital technologies can result when commercially developed food databases are used or when one standard country’s food composition database (e.g., USDA) is chosen and not tailored to the local food supply nor inclusive of ethnicities [16,20]. The design and development of fit-for-purpose country-specific food composition databases also require consideration of the foods most frequently consumed by a given population [10,11].

Therefore, the aim of the study was to determine the most popular foods and beverages (hereinafter referred to as foods) consumed in Australia in order to provide recommendations for ways to progress the design of dietary assessment technologies. The popularity of foods was determined by the frequency they are reported and by the contribution they make to daily energy intakes to ensure daily nutrient intakes are accurately captured.

## 2. Materials and Methods

### 2.1. Study Design, Setting and Participants

The Australian National Nutrition and Physical Activity Survey was a cross-sectional survey consisting of a sample from a stratified, multi-staged area of private dwellings covering 97% of the Australian population. It was the largest, most comprehensive health survey ever conducted in Australia and designed to represent the Australian population. Data were collected from Monday through to Sunday from May 2011 to June 2012. The survey was conducted by a government agency called The Australian Bureau of Statistics (ABS), and full details are published on their website [21]. Participants were selected at random from sampling fractions from each state and territory to meet this objective. Participants were aged two years and over and included one adult (aged 18 years and over) and one child (aged 2–17 years) from each household selected. The present analysis included 12,153 participants. Population demographics from this survey, including sex, age, body mass index, education level and country of birth are presented in Table 1.

### 2.2. Variables and Data Sources

The most frequently reported foods and the foods that made the largest contribution to dietary energy were the outcomes of interest. Foods were classified as discretionary or five food group. Discretionary foods were identified using the ABS discretionary food list [22] and are foods that are high in saturated fats, added sugars, salt and/or alcohol, for example, sugar sweetened beverages, cakes, sweet and savoury pastries, processed meat, sandwiches, burgers and pizzas high in saturated fat, confectionary or alcoholic beverages (e.g., beer and wine) [23]. Five food group includes: (1) grain foods (e.g., bread, breakfast cereals, rice, pasta, quinoa or barley); (2) dairy products and alternatives (e.g., milk, yoghurt, cheese, soy milk or flavoured milks); (3) fruits including whole, dried and juiced (e.g., banana, apple); (4) vegetables including legumes and 100% juices (e.g., tomato, lettuce); and (5) lean meats and alternatives (e.g., beef, pork, poultry, seafood, fish, tofu, nuts, soy beans and legumes) [23]. Five food group foods were classified as higher quality or lower quality in accordance with clarifying information regarding the quality of five food group foods by the Australian Dietary Guidelines [23]. Lower quality five food group foods included foods that fit within the five food groups but have some added sugars, salt, or are refined or processed thereby removing fibre or are otherwise higher in saturated fat. Examples include refined or low fibre grains (e.g., white bread or rice), dried and juiced fruits (e.g., sultanas and orange juice, which should only be selected sometimes), nuts and vegetables with added salt that did not otherwise classify as discretionary food in the ABS list (e.g., salted peanuts), full fat dairy products (e.g., full cream milk) or those with added sugars (e.g., flavoured milks).

Diets were assessed with two multiple pass 24 h recalls. As the second recall was only collected for a subset of the population, only the first day of the survey was used. The five-pass protocol was originally developed by the United States Department of Agriculture (USDA) and modified by the ABS and the Food Standards Australia New Zealand (FSANZ) to reflect the Australian food supply. The interview was conducted by trained ABS staff who asked participants to recall all foods consumed from midnight to midnight in the 24 h prior to the day of the interview. The AUSNUT nutrient composition was compiled from Australian nutrient composition to reflect the available foods consumed during the survey.

### 2.3. Data Analysis

Proc SURVEYFREQ was used to calculate the frequency each food that was reported and proc SURVEYMEANS was used to sum the energy for each food reported using eight-digit food codes provided by the ABS to identify individual foods. FIVDIGC food codes (minor food groups) developed by the ABS were used to further reduce the number of foods reported by the participants of the survey. Survey weights derived by the ABS were applied to ensure a representative sample of the population. Full details of the survey weights are available elsewhere (ABS User Guide [21]). All data cleaning was conducted in SAS software version 9.4 for Windows, Cary, NC: SAS Institute; 2013.

## 3. Results

A total of 4515 foods were reported as being consumed from the first day of NNPAS data. Full fat cow’s milk, beer, white rice, white bread, red wine, cola soft drink and banana were among the top 10 food and beverage sources of energy in Australians’ diets and contributed to a cumulative 10.8% of energy intake (Table 2). Dietary staples were rice, bread, breakfast cereals and milk while most other popular choices contributing to energy included were discretionary foods such as hot potato chips, sausages, ice cream and pies. The most popular mixed dish by energy contribution was pasta with beef and vegetable Bolognese, followed by lasagne.

The top 10 most frequently consumed foods and beverages were tap water, plain black tea, full fat cow’s milk, instant coffee, white sugar, reduced fat milk, banana, raw sugar and red apples, which contributed to 23% of the foods consumed by the Australian population on the day of the survey (Table 3). The most frequently consumed foods and beverages classified by food group is shown in Table 4. Different types of bread and rice were the only two grain foods reported in the top 100 foods. Common sandwich and bread toppings were also reported including vegemite, peanut butter, jam, ham, and chicken. Regarding vegetable intake, tomatoes were ranked 19th place for popularity, followed by carrots and lettuce, ranked 26th and 34th, respectively. The most popular fruits were bananas (8th), followed by apples (10th), and mandarins (28th). Dairy mainly consisted of milk and cheese. The largest category of foods was discretionary accounting for 25% of the top 100 most frequently reported foods. The most-consumed discretionary foods were sugar (5th), butter (11th), vegemite (14th), ham (15th) and cola (18th).

According to the minor food group hierarchical system (Table S1), the 10 most popular foods and beverages by energy contribution were savoury pasta or noodle dishes, full fat milk, white bread and bread rolls, rice, chicken, beef, potato products and savoury pastries such as pies and rolls, beer and battered or crumbed poultry. These foods contributed to 20.9% of total energy intake for the day of the survey. Of these 10 most popular foods, seven were either lower quality five food group foods or discretionary in nature. The 100 most popular foods, as categorised by minor food groups, contributed to 77.9% of total energy intake for Australians.

**Figure 1:**
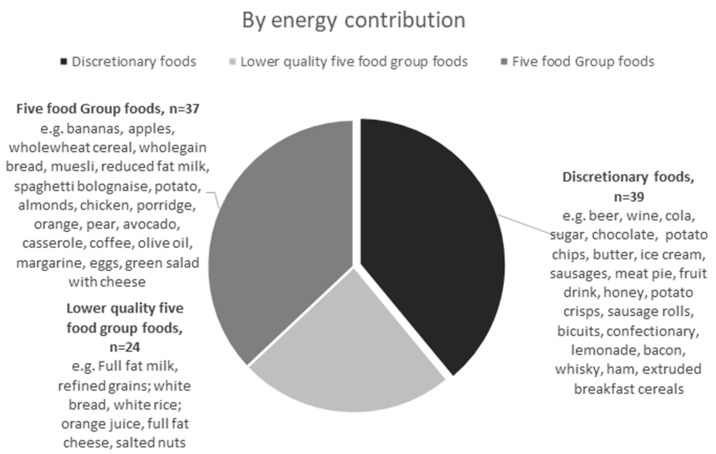
Number of the 100 most popular foods by energy contribution that were classified as five food group, lower quality five food group and discretionary foods, with a selection of the foods in each category listed.

**Figure 2:**
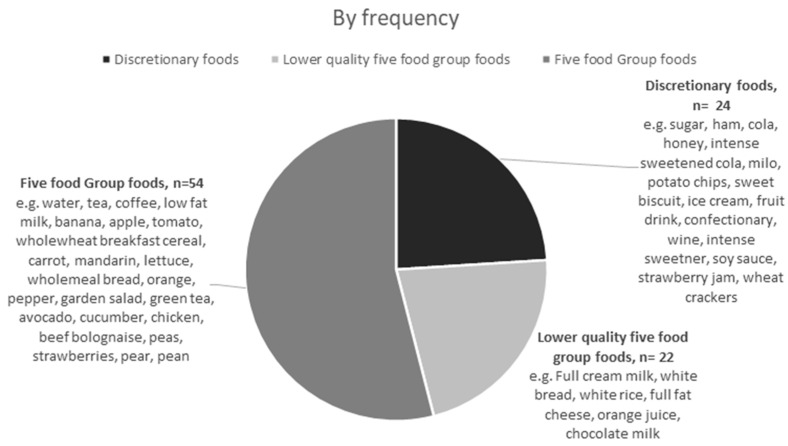
Number of the 100 most popular foods by frequency that were classified as five food group, lower quality five food group and discretionary foods, with a selection of the foods in each category listed.

**Figure 3:**
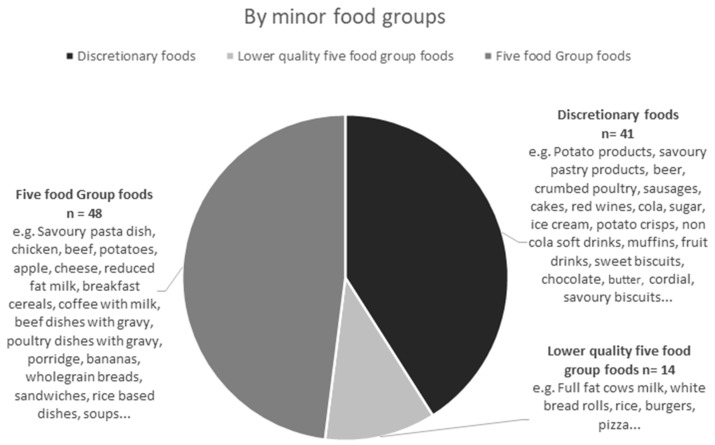
Number of the 100 most popular foods by minor food groups that were classified as five food group, lower quality five food group and discretionary foods, with a selection of the foods in each category listed.

Figure 1 depicts how discretionary foods contributed to 39 of the 100 most popular foods by energy contribution to the diet. An additional 24 foods were lower quality five food group foods, including refined grains such as white rice, white bread, full fat milk, full fat cheddar cheese and juiced fruit. Discretionary foods contributed 24 of the top 100 foods by frequency (Figure 2). The top most frequently reported discretionary foods included alcohol, sugar, butter, vegemite, ham, soft drinks, honey, biscuits, tomato sauce, potato chips, orange fruit drink, lollies, soy sauce and jam (Table 3). Based on the minor food group hierarchical system, 41% of the most popular foods were discretionary foods and an additional 11% of foods were lower quality five food group foods (Figure 3). Five food groups were more frequently reported foods (Figure 1), but discretionary foods contributed more to energy (Figure 2).

Differences between popularity by frequency of consumption and percentage energy contribution to the diet were also examined. When comparing the difference between ranking of foods by frequency to percentage energy contribution, tap water, black tea, black coffee and white sugar were ranked 1st, 3rd, 4th and 9th, respectively by frequency, but had a lower ranking by energy contribution of 4508, 167, 208 and 41. For red wine and lager or ale-style beer (alcohol 4.6% *v*/*v*), popularity by frequency of consumption was ranked 32nd and 33rd, respectively, but contribution to energy was at 2nd and 6th place. Cola soft drink on the other hand was ranked 8th place by energy contribution but was 18th place for frequency of consumption. White rice was 4th place by energy contribution but by frequency consumed ranked 29th.

## 4. Discussion

This analysis of the most-consumed foods by Australians provides data that can be used to inform innovations in digital dietary assessment and monitoring tools, such as improving search functionality or automated identification and analysis of image-based records. Furthermore, the refinement of the food databases that underpin these technologies can also be enhanced by prioritisation of these popular foods. Our data reveal that the top 100 foods (2% of the reported foods) accounted for a large proportion of the total number of foods reported by the Australian population and 53% of the foods reported in the top 100 foods, accounting for 36% of total daily energy.

Foods typifying the Australian population’s diet that require prioritisation in search returns included some five food group foods such as grain foods, apples and bananas, and discretionary foods such as hot potato chips. Our results indicate that white rice and white breads and rolls should also be prioritised in search returns, but wholegrain breads and wholewheat breakfast biscuits were still popular. These findings show similarity to the popular foods consumed in the US, though some differences also exist, for example a greater popularity of condiments (e.g., regular mayonnaise, tomato sauce/ketchup) and corn or cornmeal tortilla chips for American adults [8] compared to Australians, and the popularity of wholewheat breakfast biscuits in Australia. Therefore, these popular foods should clearly be presented as first choices when searching strings of foods within a food group in digital dietary assessment tools. For example, if cereal is searched, wholewheat breakfast biscuits or the food item’s name brand Sanitarium Weet-bix™ should appear at the top of the search.

Currently, there is a degree of complexity to discerning between hot chips and crisps (as chips is commonly used for the latter) in searches and whether to return potato chips or hot chips. Typically, search returns are for potato crisps, and hot potato chips must be discerned. Our data indicates that hot potato chips should be the first search return option. This is important, as hot chips are not only popular but also a leading source of energy in the Australian diet. The search architecture could also be improved so that a system of synonyms or fuzzy string matches should be employed and specific ways that foods are referred to in different countries implemented to enhance searchability. For example, hot chips and crisps are both called chips in Australia, but other countries may use chips, crisps, hot chips or French fries. Thus, developing food composition databases or search tools that integrate information on the frequently consumed food can improve the usability of digital dietary assessment and monitoring tools by increasing specificity for use among a certain population [11]. Moreover, it has been found that differences in gender, poverty–income ratio, race/ethnicity and body mass index status are factors that can impact what foods are most commonly consumed by different age groups [8]. Collecting such demographic and user characteristic information within digital technologies can produce food search returns that are specific to a certain sub-population and with pre-populated popular foods or with a tailored list of frequently consumed foods that can then enhance the ease of using these tools [8].

With foods such as apples, from our findings, the red variety predominates with peel on, then apple juice, red apple with peel off, and green apples. Thus, in dietary assessment technologies, rather than presenting a long list of apple types, selecting the most commonly eaten should ease the burden of recording. The food databases of some researcher-based nutrition apps have been designed so that a single entry of a generic ‘apple’ was created from the different varieties that have similar nutritional value to improve usability and relevancy of search returns [10,11]. Alternatively, search functionality can also be enhanced through modifications to in-app design features, such as the inclusion of a ‘Search-Accelerator’ function which acts as a search filter [12]. The search-accelerator function applies algorithms to assist with narrowing down a search with relevant sub-string entries and keywords [12]. This functionality could be advanced further based on our findings. For example, when a user begins to type in ‘ap’ into the search, apple may appear in the search-accelerator buttons based on prioritisation of popular foods which a user can then select. From this, the different varieties of apple would then appear in the search-accelerator buttons to which the user could then tap to quickly choose from a shorter list. Such design features would minimize users having to follow a single hierarchical structure of searching that other conventional nutrition apps may utilize. For example, when ‘apple’ is searched in the popular nutrition app MyFitnessPal, from among the first 100 search returns, 73 entries were related to the different cultivars or varieties of apple, and 27 were alternate apple-based food products (e.g., apple juice, apple pie, apple sauce). Having an excessive number of search returns is overwhelming for the user [16,17,20].

Other uses of the most popular foods are to prompt people to add forgotten foods and beverages. Previous literature has noted that up to 60% of participants may recall additional foods and beverages from these lists [24]. Common foods omitted included sweet biscuits; confectionaries and savory snack foods such as potato crisps, crackers; and fruits, vegetables and cheese [25]. These foods are among the most frequently consumed and within the top 100 foods by energy contribution observed in our analysis.

Even though nutrition apps are more convenient and accepted by users when compared to paper-based food diaries [26,27,28,29], there are still common food omissions from app records including fats and oils (54% of foods omitted), alcohol (42%) and discretionary foods and beverages (33%) [16]. As these under-reported foods align with those most commonly consumed by the population; administration of additional supplementary tools or technologies to probe or detect these foods or greater training for participants, researchers and dietitians in using these technologies would be beneficial [30]. Using continuous digital imaging methods to objectively document actual food consumption could be one way of identifying food omissions. From image-based records, the three most common foods omitted from a 24 h recall were revealed to be vegetables, fruit and confectionary items [25]. Using a digital entry app, the most commonly omitted foods were the same but confectionary outranked fruit [25].

Despite the advantages of digital imaging-based methods of dietary assessment and monitoring, coding of these images is tedious and automated image recognition is recommended [25]. Automated analysis of food images is advancing through artificial intelligence technologies [31]. Popular food items may support machine learning and identification algorithms and guide targeted search returns of suggested foods to participants or trained analysts when they are reviewing or identifying the foods in images. Having lists of frequently consumed foods might also inform processes for the verification of additions that cannot be visually detected such as types of oils and sauces used, and sugar and salt added and enhance the process of identification.

Beverages comprised five of the ten top foods and beverages consumed and five of ten for energy contribution with full fat cow’s milk topping both rankings. This is consistent with the NHANES findings where beverages, including tap water, cola, coffee, tea and milk, were among the most frequently consumed items [8]. Using digital image technology, milk was most-omitted from 24 h recall and apps but tea, sugar-sweetened beverages and alcohol were also forgotten [25]. It is therefore necessary to ensure that additions to commonly consumed foods and beverages are also prompted for by digital dietary assessment tools, given their popularity from our results. Tools to complete self-administered 24 h recalls such as the US ASA24 [32,33] and UK Intake24 [34,35] also recognise this and prompt for additions to foods and beverages such as butter on bread, milk in cereal and milk and added sugar in tea/coffee, as well as prompting for whether any beverages were consumed with a meal. Clearly prompting for beverages that include sugar in a range of forms, such as white granulated, brown and raw, is also necessary as it is highly consumed and significantly contributes to energy intakes in the population.

### 4.1. Implications for Dietitians and Public Health Interventions

Dietitians in clinical practice have a key role supporting clients to reduce disease risk and improve health outcomes through dietary changes [36,37,38]. Individualised nutrition counselling and education is particularly important given that although the most frequently reported foods were five food group foods, the largest contributors to diet in terms of energy were discretionary foods and lower quality five food group foods. Presently refined grains are selected more frequently than wholegrains. Wholegrain consumption is recommended as it reduces the risk of cardiovascular disease, type 2 diabetes, weight gain and colorectal cancer [39]. Similarly, dairy products, mostly low fat, are recommended to reduce the risk of ischemic heart disease, myocardial infarction, diabetes, hypertension and some cancers [39]. While lower fat milks were popular, full cream milk was the most commonly preferred dairy food.

Nutrition apps are useful to both patients and dietitians in nutrition care [40]. However, the majority of nutrition apps focus on tracking of calorie and nutrient intakes from food [41]. Furthermore, the option for crowd-sourced data within apps reduces accuracy [41], even despite attempts such as by MyFitnessPal to apply a green tick to the foods in the database with complete nutritional information [42]. Only one in five popular nutrition apps provided intake recommendations relating to the five food groups [41]. One integrated nutrition app platform, Easy Diet Diary Connect [43], offers more in-depth analysis and categorizes foods consumed into the five food groups. Basic assessment of variety and quality five food group foods is also present (e.g., refined vs. wholegrains and whether fruit has been consumed as fruit juice) [30]. However, discretionary foods are not directly highlighted by the Easy Diet Diary Connect platform in a clear way such as in the ABS discretionary food list. Instead, the system divides discretionary foods into their constituent ingredients and assigns them into food groups and surrogate discretionary food measures of oil equivalents, solid fat equivalents, added sugars and alcohol [44]. This method of categorization poses limitations as it risks under-representation of discretionary food intake [45]. Discretionary food intake exceeded recommended intakes and accounts for 35% of Australian adults’ energy intake in 2011/12 when it was last assessed [46]. Moreover, alcohol and its popularity has been identified in previous analyses of the NNPAS [47], and one in four people exceed recommendations for alcohol [48]. Beer and red wine were commonly consumed and major energy sources in our study.

Our findings could guide further development of the classification systems in apps so that the subcategories of discretionary foods and lower quality five food group foods can be clearly identified within apps or flagged in their associated health professional platform. This would support dietitians in their practice as they use these outputs to guide personalized nutrition interventions that improve diet quality. Furthermore, feedback algorithms and data mining are advancing and to allow for delivery of automated feedback within apps to support dietary changes [49,50,51]. These systems could be further refined so that when the most popular foods are selected, particularly if they are discretionary of lower quality five food group foods, there is the triggering of tailored and practical feedback and nutrition recommendations to encourage behaviour change.

Information technology, software and public health nutrition researchers and practitioners can draw upon these data to inform targeted digital public health campaigns and interventions. The World Health Organisation has suggested digital health interventions as a strategy for improving sustainable development goals, including population diet and nutrition [52]. Replacing higher fat dairy products with alternatives, swapping refined grains/cereals for wholegrain and reducing discretionary food consumption are key targets. Systematic reviews have revealed some evidence in support of digital interventions being efficacious for improving diet [53]. However, improving engagement with these digital dietary interventions are important and may require further innovations in intervention design [54]. Creating dietary monitoring tools that have high usability for recording food and simultaneously give targeted feedback on how to modify intake are key.

### 4.2. Strengths and Limitations

This large cross-sectional survey of the Australian population reflects the most-consumed foods in the Australian food supply. The 24 h recall method employed has been validated to provide a snapshot of what the Australian population usually eats, providing a useful data source for the most commonly reported foods in the population. The recall method of dietary assessment can also be complemented with use of food frequency questionnaires [55] to develop a more comprehensive list of frequently consumed foods. Simultaneously, these data around popular foods can also assist researchers developing or updating food frequency questionnaires to include those foods that have greatest contribution to energy consumption and that are most commonly consumed by a population [8].

It is acknowledged the data used in this study were collected 10 years ago and that dietary intakes of the population are constantly changing; no more recent population survey has been conducted. Nevertheless, this research serves as a template that may be applied to newer national datasets, and future research could examine trends over time and cross-country similarities and differences in the most popular foods by energy and frequency across populations. Furthermore, having data on the foods commonly consumed by a population can be beneficial in guiding adjustments to food composition databases to focus on these popular foods, recipes and even specific varieties or cultivars of food items over less commonly consumed foods [11]. Understanding of what foods are most popular can also allow for prioritisation of funding for direct chemical analysis of foods in updates to the nutrient values in food composition databases. Misreporting of food intakes is common in dietary surveillance and may reduce or increase the frequency of certain foods and therefore may not represent actual intakes; for example, snacks are commonly omitted from recording [56,57].

## 5. Conclusions

The findings of the most popular foods and beverages consumed in Australia can guide innovations in the design of digital tools for dietary surveillance by the development of more tailored and relevant food databases that underpin these technologies. With the most popular foods consumed also commonly under-reported or omitted in self-report records using digital dietary assessment methods, probing of these popular foods should be prioritised to improve the validity of these methods. Together with improvements to dietary assessment and monitoring technologies, dietitians and digital public health campaigns and interventions should target popular foods that could improve diet quality to enhance personalised nutrition counselling and population health.

## Figures and Tables

**Table 1:** Demographics of the population surveyed in the Australian National Nutrition and Physical Activity Survey (*n =* 12153).

Demographics	*n*	%
Sex		
Male	5702	49.7
Female	6451	50.3
**Age (years)**		
17 and under	2718	20.8
18–49	5064	46.8
50–69	2991	23.3
70 and over	1380	9.1
**Body Mass Index (BMI)**		
Underweight <18.5 kg/m^2^	3310	27.8
Normal ≥18.5 kg/m^2^–<25.0 kg/m^2^	2356	18.7
Overweight ≥25.0 kg/m^2^–<30.0 kg/m^2^	239	2.2
Obese ≥30 kg/m^2^	4273	36.3
Missing	1975	15.1
**Level of highest non-school qualification**		
Not applicable	2180	17.2
Postgraduate degree, graduate diploma/graduate certificate	770	6.1
Bachelor’s degree	1615	13.7
Advanced diploma/diploma	989	8.1
Certificate III/IV	1717	15.3
Certificate I/II	472	3.8
Certificate not further defined	74	0.6
No non-school qualification	4190	34.1
Level not determined	146	1.2
**Socio-economic index for area**		
Low (quintile 1)	2238	17.9
Middle (quintile 2–4)	7073	59.4
High (quintile 5)	2842	22.7
**Country of birth**		
Australia	9157	73.7
Main English-speaking countries (Canada, Ireland, NZ, South Africa, UK, USA)	1275	10.0
Other	1721	16.3

**Table 2:** The 100 most popular foods consumed in Australia by energy contribution.

#	Food Description	Observations (n)	Energy (%)	Cumulative Energy (%)
1	Milk, cow, fluid, regular fat (~3.5%)	2771	1.6	1.6
2	Beer, lager or ale style (alcohol 4.6% *v*/*v*)	844	1.2	2.8
3	Milk, cow, fluid, regular fat (~3.5%), not further defined	5819	1.2	4
4	Rice, white, steamed or rice cooker, no added salt	920	1.1	5.1
5	Bread, from white flour, for homemade sandwiches	1442	1	6.1
6	Wine, red	846	1	7.1
7	Bread, from white flour, commercial, toasted	1603	1	8.1
8	Soft drink, cola flavour, regular	1292	0.9	9
9	Banana, cavendish, peeled, raw	2439	0.9	9.9
10	Rice, white, boiled, no added salt	700	0.9	10.8
11	Apple, red skin, unpeeled, raw	2023	0.8	11.6
12	Breakfast cereal, whole wheat, biscuit, added vitamins B1, B2, B3 and folate, Fe and Zn	1025	0.7	12.2
13	Sugar, white, granulated or lump	4863	0.6	12.9
14	Wine, white, dry style (sugars content <1%)	499	0.6	13.5
15	Bread roll, from white flour, commercial	810	0.6	14.1
16	Chocolate, milk	643	0.5	14.6
17	Milk, cow, fluid, reduced fat (1–2%), not further defined	3703	0.5	15.2
18	Potato, chips, regular, independent takeaway outlet, cafe or restaurant, deep fried, blended oil, salted	597	0.5	15.7
19	Butter, plain, salted	2020	0.5	16.2
20	Bread, mixed grain, for homemade sandwiches	570	0.5	16.7
21	Bread, from white flour, commercial	758	0.5	17.2
22	Bread, from wholemeal flour, for homemade sandwiches	635	0.5	17.6
23	Milk, cow, fluid, reduced fat (1%)	1366	0.4	18
24	Ice cream, vanilla flavour, regular fat	570	0.4	18.5
25	Bread, from wholemeal flour, commercial, toasted	758	0.4	18.9
26	Sausage, beef, grilled, BBQed or baked	264	0.4	19.3
27	Bread, mixed grain, commercial, toasted	656	0.4	19.7
28	Cheese, cheddar, natural, plain, regular fat	766	0.4	20.2
29	Pasta dish, homemade, cooked unfilled pasta, homemade beef Bolognese sauce and added vegetables	184	0.4	20.6
30	Muesli, commercial, untoasted or natural style, added dried fruit, unfortified	276	0.4	20.9
31	Cheese, for use on sandwiches, not further defined	735	0.4	21.3
32	Pie, savoury, meat, commercial	233	0.3	21.6
33	Cheese, cheddar, natural, plain, not further defined	706	0.3	22
34	Pasta dish, homemade, cooked unfilled pasta, commercial beef Bolognese sauce	163	0.3	22.3
35	Sausage, beef, fried	213	0.3	22.6
36	Chicken, barbecued, with skin, commercial	230	0.3	23
37	Fruit drink, orange juice, commercial	568	0.3	23.3
38	Juice, orange, commercial	896	0.3	23.6
39	Potato, peeled, boiled, microwaved or steamed, drained	710	0.3	23.9
40	Lasagne, beef, with added vegetables, homemade	118	0.3	24.2
41	Sugar, raw	2384	0.3	24.4
42	Bread, from white flour, for homemade sandwiches, toasted	350	0.3	24.7
43	Porridge, rolled oats, prepared with regular fat cow’s milk	193	0.3	25
44	Beer, lager or ale style, mid strength (alcohol 3.5% *v*/*v*)	195	0.3	25.2
45	Coffee, flat white or latte, from ground coffee beans, with regular fat cow’s milk	439	0.3	25.5
46	Honey	1137	0.2	25.8
47	Biscuit, savoury, from white wheat flour, other flavours	231	0.2	26
48	Potato crisps or chips, plain, salted	300	0.2	26.3
49	Pie, savoury, meat, from frozen, baked or microwaved	171	0.2	26.5
50	Noodle, wheat, instant, flavoured, boiled, drained	134	0.2	26.7
51	Biscuit, sweet, chocolate chip, commercial	298	0.2	27
52	Bread, garlic or herb, commercial, cooked	222	0.2	27.2
53	Bread, from wheat flour, commercial, added dried fruit, toasted	252	0.2	27.4
54	Pizza, supreme, thick base, takeaway style and homemade	77	0.2	27.7
55	Pasta dish, homemade, cooked unfilled pasta, homemade beef Bolognese sauce	100	0.2	27.9
56	Sausage roll, commercial, ready to eat	146	0.2	28.1
57	Nut, almond, with or without skin, raw, unsalted	350	0.2	28.3
58	Chicken, whole, flesh, skin and fat, baked, roasted, fried, grilled or BBQed, no added fat	113	0.2	28.6
59	Porridge, rolled oats, prepared with water	292	0.2	28.8
60	Orange, peeled, raw, not further defined	780	0.2	29
61	Pear, unpeeled, raw, not further defined	467	0.2	29.2
62	Wine, white, not further defined	207	0.2	29.4
63	Avocado, raw	539	0.2	29.6
64	Biscuit, sweet, plain, commercial	576	0.2	29.8
65	Lolly, jelly varieties	514	0.2	30
66	Soft drink, lemonade, regular	441	0.2	30.2
67	Milk, cow, fluid, unflavoured, not further defined	607	0.2	30.4
68	Chicken burger, white roll, crumbed chicken breast, with lettuce and mayonnaise, fast food chain	112	0.2	30.6
69	Milk, cow, fluid, flavoured, coffee, regular fat	114	0.2	30.9
70	Potato, fries, fast food outlet, deep fried, monounsaturated oil, salted	194	0.2	31
71	Bacon, middle rasher, semi-trimmed, fried or stir-fried, no added fat	260	0.2	31.2
72	Casserole, homemade, beef and vegetables, homemade gravy	71	0.2	31.4
73	Mayonnaise, commercial, regular fat	343	0.2	31.6
74	Whisky or scotch	170	0.2	31.8
75	Cheese, cheddar, processed, regular fat	479	0.2	32
76	Bread, from wholemeal flour, commercial	306	0.2	32.2
77	Mixed drink, whisky or scotch and regular cola, commercial, pre-mixed	70	0.2	32.3
78	Potato crisps or chips, other flavours	210	0.2	32.5
79	Ham, leg, lean	1488	0.2	32.7
80	Peanut butter, smooth and crunchy, added sugar and salt	452	0.2	32.9
81	Curry, homemade, chicken & vegetable, homemade coconut milk sauce	97	0.2	33
82	Coffee, cappuccino, from ground coffee beans, with regular fat cow’s milk	310	0.2	33.2
83	Chicken, for use as a sandwich filling, not further defined	507	0.2	33.4
84	Potato crisps or chips, not further defined	231	0.2	33.5
85	Oil, olive	310	0.2	33.7
86	Potato, chips, regular, fast-food outlet, deep fried, blended oil, salted	167	0.2	33.9
87	Porridge, rolled oats, prepared with reduced fat cow’s milk	147	0.2	34
88	Beer, lager or ale style (alcohol 5% *v*/*v* & above)	143	0.2	34.2
89	Biscuit, sweet, shortbread style, commercial	260	0.2	34.4
90	Breakfast cereal, mixed grain (wheat and oat), flakes, apricot and sultana, added vitamins B1, B2, B3 and folate and Fe	174	0.2	34.5
91	Nut, peanut, without skin, roasted, with oil, salted	141	0.2	34.7
92	Milk, cow, fluid, skim (~0.15% fat), not further defined	1733	0.2	34.9
93	Margarine spread, monounsaturated (65% fat)	787	0.2	35
94	Egg, chicken, whole, hard-boiled	443	0.2	35.2
95	Egg, chicken, whole, fried, oil not further defined	320	0.2	35.3
96	Rice, white, fried with bacon or ham, egg, prawns & vegetables	76	0.1	35.5
97	Salad, garden, added cheese, no added dressing	344	0.1	35.6
98	Breakfast cereal, mixed grain (wheat, oat and corn), extruded, added vitamins B1, B2, B3, B6 and C, Ca and Fe	246	0.1	35.8
99	Mandarin, peeled, raw	925	0.1	35.9
100	Cake or cupcake, chocolate, commercial, sugar-based icing	86	0.1	36.1

**Table 3:** The 100 most frequently consumed foods by the Australian population.

#	Food Description	Weighted Frequency ^a^	Frequency ^b^	Weighted Percent (SE) ^c^
1	Water, tap	21,333,275	12,333	5.7 (0.08)
2	Tea, regular, black, brewed from leaf or teabags, plain, without milk	11,183,560	6791	3.0 (0.07)
3	Milk, cow, fluid, regular fat (~3.5%), not further defined	10,065,318	5819	2.7 (0.07)
4	Coffee, black, from instant coffee powder, without milk	9,398,347	5716	2.5 (0.06)
5	Sugar, white, granulated or lump	8,570,397	4863	2.3 (0.07)
6	Milk, cow, fluid, reduced fat (1–2%), not further defined	6,187,801	3703	1.7 (0.06)
7	Milk, cow, fluid, regular fat (~3.5%)	4,645,020	2771	1.2 (0.04)
8	Banana, cavendish, peeled, raw	4,067,173	2439	1.1 (0.03)
9	Sugar, raw	4,005,327	2384	1.1 (0.04)
10	Apple, red skin, unpeeled, raw	3,717,265	2023	1.0 (0.03)
11	Butter, plain, salted	3,341,194	2020	0.9 (0.03)
12	Milk, cow, fluid, skim (~0.15% fat), not further defined	2,876,131	1733	0.8 (0.03)
13	Bread, from white flour, commercial, toasted	2,813,117	1603	0.8 (0.04)
14	Spread, yeast, vegemite, regular	2,696,279	1542	0.7 (0.03)
15	Ham, leg, lean	2,655,824	1488	0.7 (0.03)
16	Bread, from white flour, for homemade sandwiches	2,519,940	1442	0.7 (0.03)
17	Milk, cow, fluid, reduced fat (1%)	2,455,733	1366	0.7 (0.03)
18	Soft drink, cola flavour, regular	2,289,550	1292	0.6 (0.04)
19	Tomato, common, raw	2,287,021	1225	0.6 (0.02)
20	Water, filtered	2,213,582	1180	0.6 (0.02)
21	Water, bottled, still	2,127,360	1137	0.6 (0.03)
22	Honey	2,028,067	1137	0.5 (0.03)
23	Breakfast cereal, whole wheat, biscuit, added vitamins B1, B2, B3 and folate, Fe and Zn	1,983,984	1025	0.5 (0.03)
24	Water, rainwater or tank water	1,914,018	1015	0.5 (0.04)
25	Soft drink, cola flavour, intense sweetened or diet	1,883,886	1010	0.5 (0.04)
26	Carrot, mature, peeled or unpeeled, fresh or frozen, boiled, microwaved or steamed, drained	1,852,147	958	0.5 (0.03)
27	Coffee, long black style, from ground coffee beans, without milk	1,732,645	938	0.5 (0.02)
28	Mandarin, peeled, raw	1,633,673	925	0.4 (0.02)
29	Rice, white, steamed or rice cooker, no added salt	1,602,897	920	0.4 (0.02)
30	Sauce, tomato, commercial, regular	1,585,307	905	0.4 (0.02)
31	Juice, orange, commercial	1,556,926	896	0.4 (0.02)
32	Wine, red	1,545,933	846	0.4 (0.02)
33	Beer, lager or ale style (alcohol 4.6% *v*/*v*)	1,505,790	844	0.4 (0.02)
34	Lettuce, raw, not further defined	1,505,360	823	0.4 (0.02)
35	Bread roll, from white flour, commercial	1,462,719	810	0.4 (0.02)
36	Margarine spread, monounsaturated (65% fat)	1,446,297	787	0.4 (0.02)
37	Orange, peeled, raw, not further defined	1,421,422	780	0.4 (0.02)
38	Cheese, cheddar, natural, plain, regular fat	1,418,381	766	0.4 (0.02)
39	Bread, from white flour, commercial	1,414,205	758	0.4 (0.02)
40	Bread, from wholemeal flour, commercial, toasted	1,397,106	758	0.4 (0.02)
41	Pepper, ground, black or white	1,353,441	745	0.4 (0.02)
42	Cheese, for use on sandwiches, not further defined	1,340,869	735	0.4 (0.02)
43	Salad, garden, no added dressing	1,315,693	724	0.4 (0.02)
44	Tea, green, plain, without milk	1,261,744	723	0.3 (0.02)
45	Potato, peeled, boiled, microwaved or steamed, drained	1,204,823	710	0.3 (0.03)
46	Cheese, cheddar, natural, plain, not further defined	1,200,410	706	0.3 (0.02)
47	Rice, white, boiled, no added salt	1,187,232	700	0.3 (0.02)
48	Sugar, brown	1,153,755	696	0.3 (0.02)
49	Carrot, mature, peeled or unpeeled, fresh or frozen, raw	1,140,838	666	0.3 (0.02)
50	Bread, mixed grain, commercial, toasted	1,135,784	656	0.3 (0.01)
51	Beverage base, chocolate flavour, added vitamins A, B1, B2, C, D and folate, Ca and Fe (Milo)	1,128,400	654	0.3 (0.02)
52	Chocolate, milk	1,101,879	643	0.3 (0.02)
53	Broccoli, fresh, boiled, microwaved or steamed, drained	1,093,557	636	0.3 (0.01)
54	Bread, from wholemeal flour, for homemade sandwiches	1,056,181	635	0.3 (0.02)
55	Milk, cow, fluid, unflavoured, not further defined	1,044,637	607	0.3 (0.02)
56	Potato, chips, regular, independent takeaway outlet, cafe or restaurant, deep fried, blended oil, salted	1,043,246	597	0.3 (0.02)
57	Biscuit, sweet, plain, commercial	1,014,815	576	0.3 (0.02)
58	Bread, mixed grain, for homemade sandwiches	1,013,282	570	0.3 (0.02)
59	Ice cream, vanilla flavour, regular fat	995,545	570	0.3 (0.01)
60	Fruit drink, orange juice, commercial	971,074	568	0.3 (0.02)
61	Milk, cow, fluid, skim (~0.15% fat)	970,448	565	0.3 (0.03)
62	Avocado, raw	970,196	539	0.3 (0.02)
63	Milk, cow, fluid, reduced fat (~1.5%), increased protein (~4%)	964,509	531	0.3 (0.02)
64	Cucumber, peeled or unpeeled, raw, not further defined	949,721	518	0.3 (0.02)
65	Lolly, jelly varieties	935,210	514	0.3 (0.02)
66	Chicken, for use as a sandwich filling, not further defined	903,592	507	0.2 (0.01)
67	Wine, white, dry style (sugars content <1%)	897,583	499	0.2 (0.02)
68	Margarine spread, monounsaturated (65% fat), reduced salt (sodium = 360 mg/100 g)	885,940	498	0.2 (0.02)
69	Pea, green, frozen, cooked, no added fat	876,729	497	0.2 (0.01)
70	Strawberry, raw	862,331	485	0.2 (0.01)
71	Cheese, cheddar, processed, regular fat	846,695	479	0.2 (0.02)
72	Tomato, Roma, raw	845,793	479	0.2 (0.01)
73	Pear, unpeeled, raw, not further defined	842,729	467	0.2 (0.02)
74	Peanut butter, smooth and crunchy, added sugar and salt	814,271	452	0.2 (0.02)
75	Grape, Thompson seedless or sultana, raw	771,999	449	0.2 (0.01)
76	Egg, chicken, whole, hard-boiled	765,887	443	0.2 (0.01)
77	Soft drink, lemonade, regular	739,100	441	0.2 (0.01)
78	Coffee, flat white or latte, from ground coffee beans, with regular fat cow’s milk	736,038	439	0.2 (0.02)
79	Margarine spread, polyunsaturated (70% fat)	731,355	430	0.2 (0.02)
80	Melon, watermelon, peeled, raw	730,323	426	0.2 (0.02)
81	Juice, apple, commercial	697,222	412	0.2 (0.02)
82	Intense sweetener, containing saccharin, tablet	692,685	411	0.2 (0.01)
83	Sauce, soy, commercial, regular	678,260	392	0.2 (0.01)
84	Jam, strawberry, regular	657,099	387	0.2 (0.01)
85	Lettuce, cos, raw	656,369	377	0.2 (0.01)
86	Bread, from white flour, for homemade sandwiches, toasted	644,671	350	0.2 (0.02)
87	Nut, almond, with or without skin, raw, unsalted	638,842	350	0.2 (0.02)
88	Salad, garden, added cheese, no added dressing	627,054	344	0.2 (0.01)
89	Mayonnaise, commercial, regular fat	625,584	343	0.2 (0.01)
90	Bean, green, fresh, boiled, microwaved or steamed, drained	624,961	342	0.2 (0.01)
91	Lettuce, iceberg, raw	605,750	338	0.2 (0.01)
92	Mixed vegetables, fresh or frozen, with carrot, pumpkin or sweet potato, cooked, no added fat	602,185	336	0.2 (0.01)
93	Cauliflower, fresh or frozen, boiled, microwaved or steamed, drained	601,030	331	0.2 (0.01)
94	Tomato, raw, not further defined	578,807	327	0.2 (0.01)
95	Apple, red skin, peeled, raw	572,491	323	0.2 (0.01)
96	Egg, chicken, whole, fried, oil not further defined	569,730	320	0.2 (0.01)
97	Coffee, cappuccino, from ground coffee beans, with regular fat cow’s milk	568,643	310	0.2 (0.01)
98	Oil, olive	566,981	310	0.2 (0.02)
99	Apple, green skin, unpeeled, raw	554,978	308	0.1 (0.01)
100	Biscuit, savoury, from white wheat flour, plain snack cracker style	550,432	307	0.1 (0.01)

^a^—the sum of the estimated weighted frequencies for all type foods or beverages reported in one day of the survey, with survey weights applied; ^b^—the frequency that a food or beverages was reported without dietary weights; ^c^—derived from the weighted frequency of the individual food or beverage divided by the total weighted frequency of all foods and beverages (n = 373,320,769); SE = standard error.

**Table 4:** The 100 most frequently consumed foods by the Australian population by food group.

Grains	Dairy	Meat	Fruit	Vegetables, Herbs and Spices	Discretionary	Beverages	Fats and Oils
Bread, from white flour, commercial, toasted	Milk, cow, fluid, regular fat (~3.5%), not further defined	Chicken, for use as a sandwich filling	Banana, cavendish, peeled, raw	Tomato, common, raw	Sugar, white, granulated or lump	Water, tap	Margarine spread, monounsaturated (65% fat)
Bread, from white flour, for homemade sandwiches	Milk, cow, fluid, reduced fat (1–2%), not further defined	Egg, chicken, whole, hard-boiled	Apple, red skin, unpeeled, raw	Carrot, mature, cooked	Sugar, raw	Tea, regular, black, from leaf or teabags, plain, without milk	Margarine spread, monounsaturated (65% fat), reduced salt
Breakfast cereal, whole wheat, biscuit	Milk, cow, fluid, regular fat (~3.5%)	Nut, almond, raw, unsalted	Mandarin, peeled, raw	Lettuce, raw	Butter, plain, salted	Coffee, black, instant powder, without milk	Margarine spread, polyunsaturated (70% fat)
Rice, white, steamed or rice cooker	Milk, cow, fluid, skim (~0.15% fat)	Egg, chicken, whole, fried	Juice, orange, commercial	Pepper, ground, black or white	Spread, yeast, vegemite, regular	Water, filtered	Mayonnaise, commercial, regular fat
Bread roll, from white flour, commercial	Milk, cow, fluid, reduced fat (1%)		Orange, peeled, raw	Salad, garden, no added dressing	Ham, leg, lean	Water, bottled, still	Oil, olive
Bread, from white flour, commercial	Cheese, cheddar, natural, plain, regular fat		Strawberry, raw	Potato, peeled, cooked without fat	Soft drink, cola flavour, regular	Water, rainwater or tank water	
Bread, from wholemeal flour, commercial, toasted	Cheese, for use on sandwiches, not further defined		Pear, unpeeled, raw	Carrot, mature, raw	Honey	Coffee, long black style, from ground coffee beans, without milk	
Rice, white, boiled, no added salt	Cheese, cheddar, natural, plain, not further defined		Grape, Thompson seedless or sultana, raw	Broccoli, fresh, boiled, microwaved or steamed, drained	Soft drink, cola flavour, intense sweetened or diet	Tea, green, plain, without milk	
Bread, mixed grain, commercial, toasted	Chocolate, milk		Melon, watermelon, peeled, raw	Avocado, raw	Sauce, tomato, commercial, regular	Coffee, flat white or latte, from ground coffee beans, with regular fat cow’s milk	
Bread, from wholemeal flour, for homemade sandwiches	Milk, cow, fluid, unflavoured, not further defined		Juice, apple, commercial	Cucumber, peeled or unpeeled, raw, not further defined	Wine, red	Coffee, cappuccino, from ground coffee beans, with regular fat cow’s milk	
Bread, mixed grain, for homemade sandwiches	Milk, cow, fluid, skim (~0.15% fat)		Apple, red skin, peeled, raw	Pea, green, frozen, cooked, no added fat	Beer, lager or ale style (alcohol 4.6% *v*/*v*)		
Bread, from white flour, for homemade sandwiches, toasted	Milk, cow, fluid, reduced fat (~1.5%), increased protein (~4%)		Apple, green skin, unpeeled, raw	Tomato, Roma, raw	Sugar, brown		
	Cheese, cheddar, processed, regular fat			Lettuce, cos, raw	Beverage base, chocolate flavour (Milo)		
				Salad, garden, added cheese, no added dressing	Potato, chips, regular, independent takeaway outlet, cafe or restaurant, salted		
				Bean, green, fresh, boiled, microwaved or steamed, drained	Biscuit, sweet, plain, commercial		
				Lettuce, iceberg, raw	Ice cream, vanilla flavour, regular fat		
				Mixed vegetables, fresh or frozen, with carrot, pumpkin or sweet potato, cooked, no added fat	Fruit drink, orange juice, commercial		
				Cauliflower, fresh or frozen, cooked without fat	Lolly, jelly varieties		
				Tomato, raw	Wine, white, dry style		
					Peanut butter, smooth and crunchy, added sugar and salt		
					Soft drink, lemonade, regular		
					Intense sweetener		
					Sauce, soy		
					Jam, strawberry, regular		
					Biscuit, savoury, from white wheat flour, plain snack cracker style		

## Data Availability

Data are available upon request from the Australian Bureau of Statistics https://www.abs.gov.au/statistics/microdata-tablebuilder/available-microdata-tablebuilder, accessed on 15 May 2018.

## Supplementary Materials

The following supporting information can be downloaded at: https://www.mdpi.com/article/10.3390/nu14224822/s1, Table S1: the 100 most popular foods in Australia by energy contribution when categorized by minor food groups.
