# Supplementary material for: Using Popular Foods Consumed to Inform Development of Digital Tools for Dietary Assessment and Monitoring
Source: Nutrients. 2022 Nov 14;14(22):4822. doi: 10.3390/nu14224822 (PMC9698260; doi:10.3390/nu14224822)
Supplement: Supplementary file 1 [file nutrients-14-04822-s001.zip › nutrients-1902509-supplementary.pdf]

Table S1: The 100 most popular foods in Australia by energy contribution when categorised by minor food groups

#	Minor food groups	Observations (n)	Energy (%)	Cumulative Energy (%)
1	Savoury pasta/noodle and sauce dishes, saturated fat ≤5 g/100 g	1626	3.2	3.2
2	Milk, cow, fluid, regular whole, full fat	8710	2.8	6.0
3	Breads, and bread rolls, white, mandatorily fortified	3948	2.7	8.6
4	Rice and rice grain fractions	1836	2.2	10.8
5	Chicken	2204	2.1	12.9
6	Beef	1452	1.7	14.6
7	Potato products	1674	1.6	16.2
8	Savoury pastry products, pies, rolls and envelopes	1066	1.6	17.8
9	Beers, > 3.5% alcohol	1113	1.5	19.4
10	Poultry crumbed, battered, meatloaf or patty type with cereal and/or vegetables	969	1.5	20.9
11	Breads, and bread rolls, white, not stated as to fortification	1914	1.4	22.3
12	Burgers, saturated fat ≤5 g/100 g	634	1.4	23.7
13	Pizza, saturated fat ≤5 g/100 g	550	1.4	25.1
14	Sausage, saturated fat content >5 g/100g	792	1.2	26.3
15	Cakes and cake mixes, other types	820	1.2	27.5
16	Potatoes	2041	1.1	28.6
17	Apples	3010	1.1	29.7
18	Cheese, hard cheese ripened styles	2440	1.1	30.9
19	Milk, cow, fluid, reduced fat, <2 g/100g	5662	1.1	32.0
20	Wines, red (including sparkling varieties and rose styles)	899	1.1	33.0
21	Breakfast cereal, mixed grain, with fruit and/or nuts	784	1.1	34.1
22	Soft drinks, cola	1467	1.0	35.1
23	Coffee beverage, prepared with milk or milk substitute	2115	1.0	36.2
24	Wines, white (including sparkling varieties)	875	1.0	37.2
25	Sugar	8052	1.0	38.2
26	Breakfast cereal, wheat based, fortified, sugars ≤20 g/100g	1586	1.0	39.2
27	Beef dishes with gravy, sauce or vegetables	623	1.0	40.1
28	Poultry dishes, with gravy, sauce or vegetables	550	1.0	41.1
29	Porridge style, oat based	899	0.9	42.0
30	Bananas	2446	0.9	42.9
31	Ice cream, tub varieties, fat content >10 g/100 g	1039	0.9	43.8
32	Breads, and bread rolls, mixed grain, mandatorily fortified	1347	0.9	44.7
33	Sandwiches and filled rolls, saturated fat ≤5 g/100 g	459	0.9	45.6

34	Breads, and bread rolls, wholemeal and brown, mandatorily fortified	1441	0.9	46.5
35	Lamb and mutton	687	0.9	47.3
36	Savoury rice-based dishes, saturated fat ≤5 g/100 g	442	0.8	48.2
37	Potato crisps	1004	0.8	49.0
38	Soft drinks, non-cola	1376	0.8	49.7
39	Muffins, cake type, and muffin mixes	359	0.8	50.5
40	Fruit drinks (ready to drink or made from concentrate)	1401	0.7	51.2
41	Sweet biscuits, plain or flavoured including short bread varieties	1722	0.7	52.0
42	Chocolate (plain, unfilled varieties)	952	0.7	52.7
43	Fruit juices, commercially prepared	1881	0.7	53.4
44	Finfish, battered or crumbed	441	0.7	54.1
45	Chocolate-based confectionery with other fillings or additions	894	0.7	54.8
46	Soup containing meat, poultry or seafood	666	0.7	55.5
47	Other nuts and nut products and dishes	929	0.7	56.2
48	Cakes and cake mixes, chocolate	378	0.7	56.9
49	Pork	551	0.7	57.5
50	Finfish, fresh, frozen	575	0.6	58.2
51	Butter	2449	0.6	58.8
52	Eggs, chicken	1467	0.6	59.4
53	Cordials, made from concentrate	857	0.6	60.0
54	Breads, and bread rolls, mixed grain, not stated as to fortification	719	0.6	60.6
55	Breads, and bread rolls, wholemeal, not stated as to fortification	796	0.6	61.1
56	Salads, vegetable based	2130	0.6	61.7
57	Pasta and noodles, wheat based, other than instant noodles	467	0.6	62.3
58	Milk, coffee/chocolate flavoured and milk-based drinks, full fat	387	0.5	62.8
59	Egg dishes, savoury	592	0.5	63.3
60	Savoury biscuits, wheat based, plain, energy >1800 kJ per 100 g	861	0.5	63.9
61	Poultry dishes, with gravy, sauce or vegetables, added pasta, noodles or rice	243	0.5	64.3
62	Flat breads (e.g. Pita bread), wheat based	554	0.5	64.8
63	Breakfast cereal, mixed grain, with fruit and/or nuts, fortified	557	0.5	65.3
64	Pizza, saturated fat >5 g/100 g	182	0.4	65.7
65	Lollies and other confectionery, sugar sweetened	1239	0.4	66.2
66	Soup, vegetable only	619	0.4	66.6
67	Instant noodles and noodle products, wheat based	291	0.4	67.0
68	Sweet breads, buns and scrolls, uniced, unfilled	456	0.4	67.5

69	Bacon	644	0.4	67.9
70	Other fruit	1794	0.4	68.3
71	Potato mixed dishes	444	0.4	68.7
72	Mayonnaise and cream-style dressings, full fat	853	0.4	69.0
73	Beers, 1.15- 3.5% alcohol, reduced alcohol / light	346	0.4	69.4
74	Sweet biscuits, chocolate-coated, chocolate chip	536	0.4	69.8
75	Cheese, not further defined	770	0.4	70.2
76	Monounsaturated margarine spreads, fat content ≥65 g/100g	1774	0.4	70.5
77	Packed finfish	652	0.4	70.9
78	Sweet biscuits, cream-filled	521	0.3	71.2
79	Chocolate-based confectionery with nut fillings or additions	436	0.3	71.6
80	Slices, biscuit and cake-type	263	0.3	71.9
81	Lamb or mutton dishes with gravy, sauce or vegetables	160	0.3	72.2
82	Salads, vegetable based, added meat, fish or eggs	534	0.3	72.6
83	Yoghurt, flavoured or added fruit, reduced fat	587	0.3	72.9
84	Savoury biscuits, wheat based, plain, energy ≤1800 kJ per 100 g	760	0.3	73.2
85	Yoghurt, flavoured or added fruit, full fat	557	0.3	73.5
86	Mixed nuts or nuts and seeds	270	0.3	73.8
87	Burgers, saturated fat >5 g/100 g	177	0.3	74.1
88	Vegetables and sauce	274	0.3	74.5
89	Mixtures of two or more vegetables	1251	0.3	74.8
90	Beef, crumbed, battered, meatloaf or patty type with cereal and/or vegetables	298	0.3	75.1
91	Beef dishes, added pasta, noodles or rice	160	0.3	75.4
92	Spirits	278	0.3	75.6
93	Pears	669	0.3	75.9
94	Savoury pastry products, pies, rolls and envelopes, fried	204	0.3	76.2
95	Honey and sugar syrups	1269	0.3	76.5
96	Milk, cow, fluid, skim, non-fat	2326	0.3	76.8
97	Muesli and cereal style bars, with fruit and/or nuts	443	0.3	77.1
98	Pre-mixed drinks, cola- or energy-drink based	121	0.3	77.4
99	Cakes and cake mixes, sponge	237	0.3	77.7
100	Other fruiting vegetables	1815	0.3	77.9
